# The emergence of carbapenem resistance in ESBL-producing *Escherirchia coli* O25B-ST131 strain from community acquired infection in Kuwait

**DOI:** 10.1186/1753-6561-5-S6-O27

**Published:** 2011-06-29

**Authors:** AA Dashti, L Vali, MM Jadaon, S El-Shazly, SG Amyes

**Affiliations:** 1 Medical Laboratory Sciences, Kuwait University, Kuwait; 2 Molecular Chemotherapy, University of Edinburgh, UK

## Introduction / objectives

In this study we investigated a multi-drug resistant *E.coli* recovered from ascetic fluid of a haemodialysis patient with community-onset urinary tract infection from Al-Amiri hospital in Kuwait. The patient was suffering from advanced liver disease with portal hypertension and multiple current inter abdominal abscesses.

## Methods

Antimicrobial susceptibility was determined by Vitek2, Microscan, disc diffusion, E-test & double disc method against antibiotics. PCR& sequencing were performed forO25pabBspe**,***pabB*, *trpA*, *chuA*, *yjaA* TSPE4, *bla*SHV, *bla*TEM, *bla*CTX-M15, *bla*_OXA-1-like_, *aac*(*6*′)*-Ib-cr*, *tet*(A), *tet*(B), *gyr*A, *par*C, plasmid mediated *qnr*A, *qnr*B, *qnr*S, IMP, SPM, VIM, OXA-48, NDM, KPC and classes 1and 2 integrons.

## Results

The isolate was confirmed as *E. coli* O25b-sequence type (ST) 131 clone of B2 phylogenetic group. The isolate was resistant to all antibiotics tested except sulfamethoxazole, trimethoprim and nitrofurantoin and E-test confirmed that it is highly resistant to meropenem, imipenem, ciprofloxacin, cefotaxime and ceftazidime with MIC values of >16 mg/l, 32 mg/l, >64 mg/l, 32 mg/l & >32mg/l respectively. PCR detected the expected sizes of the amplified resistance genes, and DNA sequencing confirmed that TEM-1, the novel SHV-122 GeneBank (GQ290211), CTX-M-15, OXA-1, variant *aac*(*6*′)*-Ib-cr*, *tet*(A) genes, VIM and KPC were present and it was found to carry a class 1 integron. No mutation was found in *gyr*A but in ParC a mutation at 520 G to C, with amino acid change 174 Val (GTC) to Leu (CTC) was detected. QnrA, B, S and integron 2 were not present.

## Conclusion

This is the first report of the emergence and the detection of a multiple antibiotic resistant *E. coli* O25b-sequence type (ST)131 containing 2 carbapenemase genes in Kuwait.

## Disclosure of interest

None declared.

